# Phytochemical Profiles and Cytotoxic Activity of *Bursera fagaroides* (Kunth) Engl. Leaves and Its Callus Culture

**DOI:** 10.3390/plants13121622

**Published:** 2024-06-12

**Authors:** Nancy Pérez-Mejía, María Luisa Villarreal, Jessica Nayelli Sánchez-Carranza, Leticia González-Maya, Manasés González-Cortazar, Anabel Ortíz-Caltempa, Laura Alvarez

**Affiliations:** 1 Centro de Investigación en Biotecnología, Universidad Autónoma del Estado de Morelos, Avenida Universidad 1001, Col. Chamilpa, Cuernavaca C. P. 62209, Mexico; maria.perezme@uaem.edu.mx (N.P.-M.); luisav@uaem.mx (M.L.V.); 2 Facultad de Farmacia, Universidad Autónoma del Estado de Morelos, Avenida Universidad 1001, Col. Chamilpa, Cuernavaca C. P. 62209, Mexico; jessica.sanchez@uaem.mx (J.N.S.-C.); letymaya@uaem.mx (L.G.-M.); 3 Centro de Investigación Biomédica del Sur, IMSS, Calle República Argentina No. 1, Col. Centro, Xochitepec C. P. 62790, Mexico; gmanases@hotmail.com; 4 Centro de Investigaciones Químicas, Universidad Autónoma del Estado de Morelos, Avenida Universidad 1001, Col. Chamilpa, Cuernavaca C. P. 62209, Mexico

**Keywords:** *Bursera fagaroides*, callus culture, leaves, scopoletin, cytotoxicity, chemical profiles

## Abstract

*Bursera fagaroides*, popularly used in México, possesses bioactive lignans. These compounds are low in the bark, and its extraction endangers the life of the trees. The aim of the present investigation was to search for alternative sources of cytotoxic compounds in *B*. *fagaroides* prepared as leaves and in vitro callus cultures. The friable callus of *B. fagaroides* was established using a combination of plant growth regulators: 4 mgL^−1^ of 2,4-dichlorophenoxyacetic acid (2,4-D), 1 mgL^−1^ Naphthaleneacetic Acid (NAA) and 1 mgL^−1^ Zeatin. The maximum cell growth was at day 28 with a specific growth rate of μ = 0.059 days^−1^ and duplication time td = 11.8 days. HPLC quantification of the dichloromethane callus biomass extract showed that Scopoletin, with a concentration of 10.7 µg g^−1^ dry weight, was the main compound inducible as a phytoalexin by the addition of high concentrations of 2,4-D, as well as by the absence of nutrients in the culture medium. In this same extract, the compounds γ-sitosterol and stigmasterol were also identified by GC-MS analysis. Open column chromatography was used to separate and identify yatein, acetyl podophyllotoxin and 7′,8′-dehydropodophyllotoxin in the leaves of the wild plant. Cytotoxic activity on four cancer cell lines was tested, with PC-3 prostate carcinoma (IC_50_ of 12.6 ± 4.6 µgmL^−1^) being the most sensitive to the wild-type plant extract and HeLa cervical carcinoma (IC_50_ of 72 ± 5 µgmL^−1^) being the most sensitive to the callus culture extract.

## 1. Introduction

*Bursera fagaroides* (Kunth) Engl. belongs to the Burseraseae family and is commonly known as “aceitillo”, “copalillo”, “copal”, “cuajiote amarillo”, “palo del diablo” or “papelillo”. It is an aromatic tree or shrub that grows to between 0.5 and 8 m high and is dioecious. It has generally imparipinnate leaves, its branches have abundant transparent resin and its trunk is green. It is distributed from the southwest of the United States of America to the south of México [1,2,3]. Its bark is yellowish gray, and it is used in traditional medicine to relieve inflammation, skin tumors and warts [4]. The chemical study of *B. fagaroides* over the years has included the characterization of 19 lignans structures classified into four groups: eight are aryltetralins: podophyllotoxin (**1**), β-peltatin-A-methylether (**2**), 5′-desmethoxy-β-peltatin-A-methylether (**3**), desoxypodophyllotoxin (**4**), acetyl podophyllotoxin (**5**), morelensine (**6**), burseranin (**7**) and acetylpicropodophyllotoxin (**8**); three are dibenzylbutyrolactone: desmethoxy-yatein (**9**), yatein (**10**) and hinoquinin (**11**); three are aryldihydronaphtalene: 7′,8′-dehydropodophyllotoxin (**12**), 7′,8′-dehydroacethyl podophyllotoxin (**13**) and 7′,8′-dehydro trans-p-cumaroylpodophyllotoxin (**14**); and five are lignan dibenzylbutanes: 9-acetyl-9′-pentadecanoyldihydroclusin (**15**), 2,3-desmethoxy-secoisolintetralin monoacetate (**16**), dihydroclusin diacetate (**17**), 2,3-demethoxy-secoisolintetralin diacetate (**18**) and dihydroclusin mono acetate (**19**) [5,6,7]. These compounds have been isolated from the bark using solvents with different polarities [5,6,8,9,10], and these compounds present important cytotoxic activity with IC_50_ values ranging from 12 µgmL^−1^ (burseranin, **7**) to 4.43 × 10^−6^ µgmL^−1^ (β-peltatin-A-methylether, **2**). Some have been evaluated by their antitumor effect (**2** and **3**). It has been demonstrated that some of them (**1**, **2**, **5** and **9**) promote mitotic arrest, delay cell migration and disrupt microtubules [5,6,7,8,9,10,11]. Their extraction and purification have been carried out from the bark and require large amounts of plant biomass. However, the high percentage of empty seeds, low germination rates and the difference in leaflet shape and size between shrubs and trees limit biomass production in their natural populations [12,13]. Furthermore, given the magnitude of tropical deforestation in recent decades, the fact that about 70% of tropical hardwood forests have been drastically lost [14,15] and that it is also one of the most threatened ecosystems in the country, it is necessary to seek alternatives for its extraction.

Plant cell culture is a biotechnological tool that allows the cultivation of plant explants in appropriate culture media [16,17]. Plant material can be used to obtain callus cultures that can be scaled up in liquid systems, allowing controlled production of secondary metabolites independent of environmental conditions. For several years, there has been a particular interest in the establishment of calluses from various plants for mass propagation or production of compounds of interest to be scaled up in suspension cultures [18,19,20,21,22]. However, research on in vitro culture of the Burseraceae family is limited, as described below.

In vitro cultures of some species of the Burseraceae family have been established, and compounds of biological interest have been obtained, such as guggulsterone (a plant sterol that inhibits the growth of a wide variety of tumor cells and induces apoptosis [23]). This compound was synthesized in the callus of *Commiphora wightii*, grown in all treatments with 2,4-D and kinetin [24]. On the other hand, callus cultures and cell suspensions of *Commiphora gileadensis* can be used to produce secondary metabolites (quercetin, 10-hydroxycamptothecin, floginax and puromycin) with antimicrobial and cytotoxic effects [25]. Another study was on the production of boswellic acid (anti-inflammatory and anti-arthritic agent) in calluses obtained from root, stem, cotyledon and leaf explants of *Boswellia serrata* [26].

*Bursera fagaroides* is a plant with medicinal, antitumor and cytotoxic properties due to the different components it synthesizes [4,5,6,7,8,9,10,11]. The traditional method of extraction or chemical synthesis is performed using the bark, but this practice is considered unsustainable due to high production costs, low yields and severe damage to the plant. Therefore, it is necessary to look for attractive alternatives for its production. The aim of this work was to study the metabolism present in friable callus cultures of *Bursera fagaroides* and to compare their production with that of the leaves from the wild plant.

## 2. Results

### 2.1. Obtaining Leaves from the Cultivation of Cuttings

**Figure 1:**
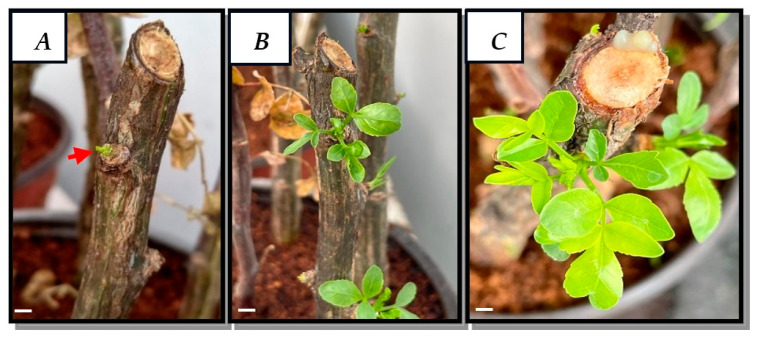
Cultivation of *B. fagaroides* cuttings at (**A**) 12, (**B**) 25 and (**C**) 70 days. Bar = 0.5 cm.

Cuttings grown in pots with coconut fiber and maintained under greenhouse conditions showed good development and growth, as shown in Figure 1. At 12 days, the first leaf buds of approximately 0.5 cm in length developed (Figure 1A), and, at 25 and 70 days, healthy leaves of between 2 and 3 cm in length developed (Figure 1B,C).

### 2.2. Callus Induction

**Figure 2:**
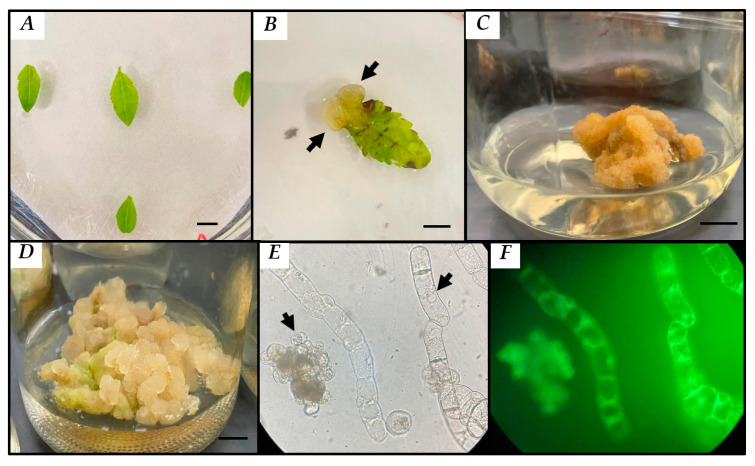
Callus induction and development from *B. fagaroides* leaf explants in photoperiod. (**A**) Disinfection of explants, (**B**) dedifferentiated callus after 30 days in MS medium supplemented with 2,4-D:Kinetin at a ratio of 2:2 mgL^−1^, (**C**) callus culture in MS medium supplemented with 2,4-D:Kinetin at a ratio of 2:2 mgL^−1^ after the subcultures, (**D**) callus culture at 30 days in B5 medium with 2,4-D:NAA:Zeatin at a ratio of 4:1:1 mgL^−1^, (**E**,**F**) callus morphology and cellular viability in B5 medium with 2,4-D:NAA:Zeatin at a ratio of 4:1:1 mgL^−1^, respectively, at 30 days of culture. Bar = 0.5 cm and 40× objective.

The disinfection process allowed us to obtain 90% of the leaves that were free of contamination in the different treatments and conditions tested (Figure 2A). Of all the concentrations tested, the MS medium with a combination of 2 mgL^−1^ of 2,4-D and 2 mgL^−1^ of Kinetin had 86.67% dedifferentiated cells, as summarized in Table 1. However, as shown in Figure 2B, there was a higher proliferation of cells at the base of the explant after 30 days. In addition, a more compact callus developed, with a green coloration that, after the subcultures, oxidized and turned brown (Figure 2C), so they were transferred to B5 medium using 4 mgL^−1^ of 2,4-D, 1 mgL^−1^ of NAA and 1 mgL^−1^ of Zeatin. As shown in Figure 2D, this process promoted a better callus formation and growth response with more friable characteristics, and individual cells were observed to be predominantly of elongated morphology; however, circular cells were also observed (Figure 2E), with 98% cell viability (Figure 2F).

### 2.3. Growth Kinetics, Percentage of Viability and Appearance of the Callus Culture

**Figure 3:**
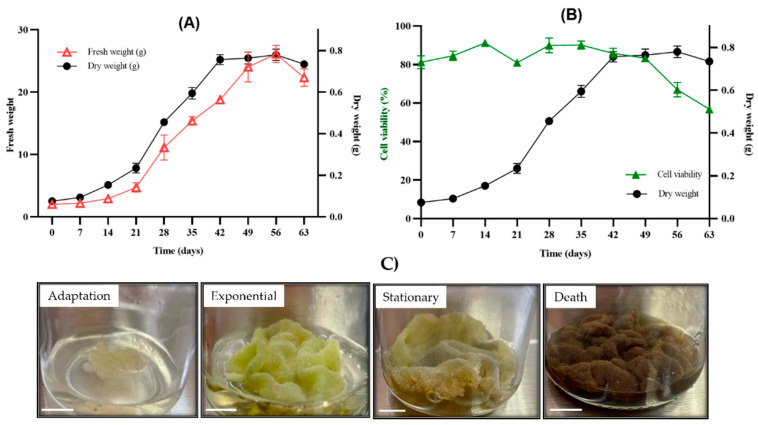
(**A**) Growth curve, (**B**) percentage cellular viability and (**C**) appearance of callus culture from *B. fagaroides*. Bar = 0.5 cm.

The growth curve of the callus culture with the weights of the fresh and dry biomass is shown in Figure 3A. The growth kinetics allowed a sigmoidal behavior to be obtained over 63 days, in which the adaptation phase was from days 1 to 14, the exponential phase was from days 14 to 42, reaching a specific growth rate of µ = 0.059 days^−1^ and a td = 11.8 days, the stationary phase was from days 42 to 56 and, finally, the death phase was seen from days 56 to 63. It should be noted that the highest growth rate (GI) was observed on day 28.

The viability of callus culture is shown in Figure 3B and cell appearance of callus in Figure 3C. The culture started with 81% cell viability, which increased slightly to 91% during the adaptation phase (days 0 to 14). Here, the cultures presented a friable consistency with a light yellow coloration. Later in the exponential phase (days 21 to 42), the callus took on a coloration that went from light green to dark green with an average of between 80 and 90% viability. Then, in the stationary and death phases, the cultures turned dark brown, reaching 56% viability.

Once the callus cultures were established and the kinetic parameters were determined, their chemical profiles were compared with the chemical content of the wild leaves of *B. fagaroides*. For this purpose, their organic extracts were simultaneously obtained with CH_2_Cl_2_ and analyzed by GC-MS and HPLC. The main results of this analysis are described below.

### 2.4. Chemical Profile of B. fagaroides Callus Culture

**Figure 4:**
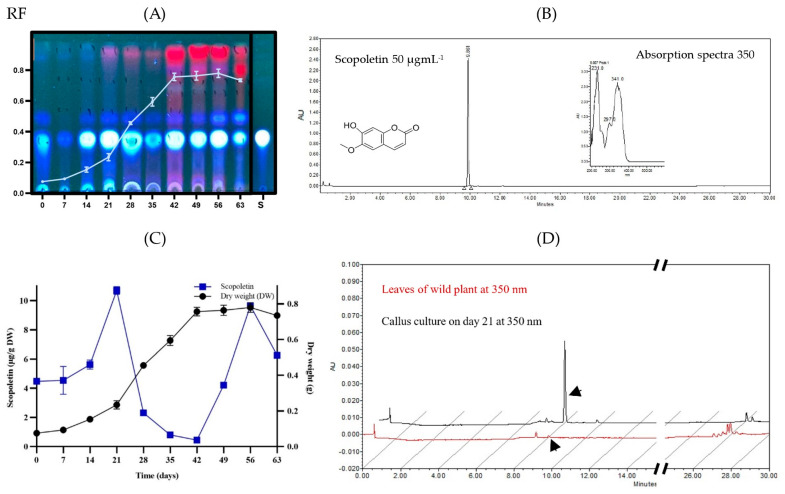
(**A**) TLC qualitative analysis of callus extracts on different days of culture, (**B**) chromatogram of scopoletin standard at 350 nm (50 mgmL^−1^), (**C**) production curve of scopoletin (values are expressed as the mean ± SD of three independent experiments) and (**D**) production of scopoletin in callus extract and wild plant leaf of *B. fagaroides*.

The CH_2_Cl_2_ extract of the dry plant biomass from each of the days of *B. fagaroides* growth kinetics was first analyzed qualitatively by TLC (Figure 4A). In the adaptation phase, minimal production of secondary metabolites was observed compared to in the stationary phase, where low-polarity compounds associated with cell growth were observed. However, in the stationary phase, their production increased considerably, maintaining the maximum concentration from days 42 to 56. This analysis suggests that the callus culture of this species synthesizes compounds of lower polarity in the stationary phase, possibly in response to the absence of nutrients in the medium. It should be noted that, during the culture, one of the major compounds was the one that presented an Rf equal to 0.35, with a bright blue coloration observed at 360 nm, which was obtained constantly from day 14 onwards and was maintained until the end of the exponential phase, decreasing its presence considerably in the cell death phase. This molecule was compared with the scopoletin standard (TLC-S). To confirm its presence, first, a commercial standard was used and analyzed by HPLC at 360 nm, which presented an RT of 10.27 min (Figure 4B), and then each of the spots of the callus culture was also analyzed. Their concentration expressed in μg of compound per g of dry biomass was quantified. As can be seen in Figure 4C, on day 21, corresponding to the beginning of the exponential phase, the first production peak of the metabolite (10.7 μg g^−1^) was observed, which decreased as the cell was involved in growth and cell division. On the other hand, when the cells entered the stationary phase, its production increased again, meaning that, at the end of this phase (day 56), there was a second production peak (9.7 μg g^−1^), possibly in response to the absence of nutrients.

Finally, the scopoletin production of the extract from the callus culture was compared against that of the extract from the leaves of the wild plant, and it was observed that the accumulation of scopoletin in the wild plant was low with respect to the callus culture (Figure 4D).

After analyzing the production of scopoletin, the chemical profile of the callus biomass was analyzed. Table S1 and Figure S2 (Supporting Information) display the GC-MS analysis of the dichloromethane extract from the biomass of the *B. fagaroides* callus culture. This analysis indicated the presence of ten metabolites, of which four (9.4%) were terpenes, three (7.5%) were fatty acids and three were sterols (33.8%). The predominant metabolites were the sterol γ-sitosterol, with a retention time of 35.11 min and a relative abundance of 32%; the triterpene betulinaldehyde (RT = 37.83 min and relative abundance = 5.9%); and 7-Methyl-Z-tetradecen-1-ol acetate (RT = 23.58 min and relative abundance = 3%).

**Figure 5:**
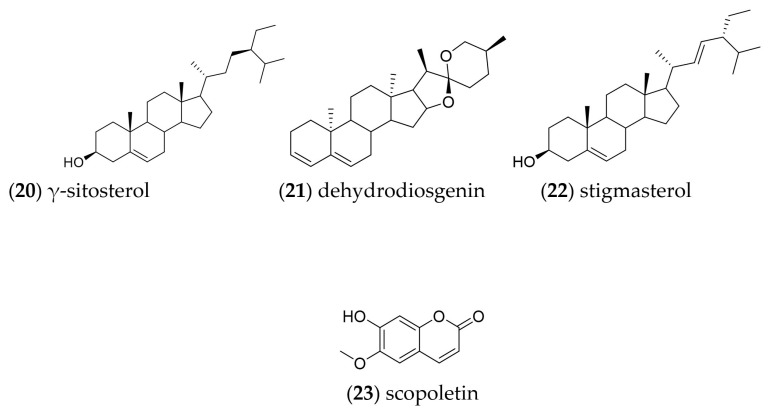
Chemical structures of compounds identified by GC-MS and isolated by CC from *B. fagaroides* callus culture.

Open column fractionation of the callus culture dichloromethane extract allowed the identification of the following compounds by gas chromatography coupled to mass spectrometry: gamma-sitosterol (**20**), dehydrodiosgenin (**21**), stigmasterol (**22**) and scopoletin (**23**) (Figure 5). Chromatograms are displayed in Figures S3–S5 (Supporting Information).

### 2.5. Chemical Profile of Wild B. fagaroides Leaves

**Figure 6:**
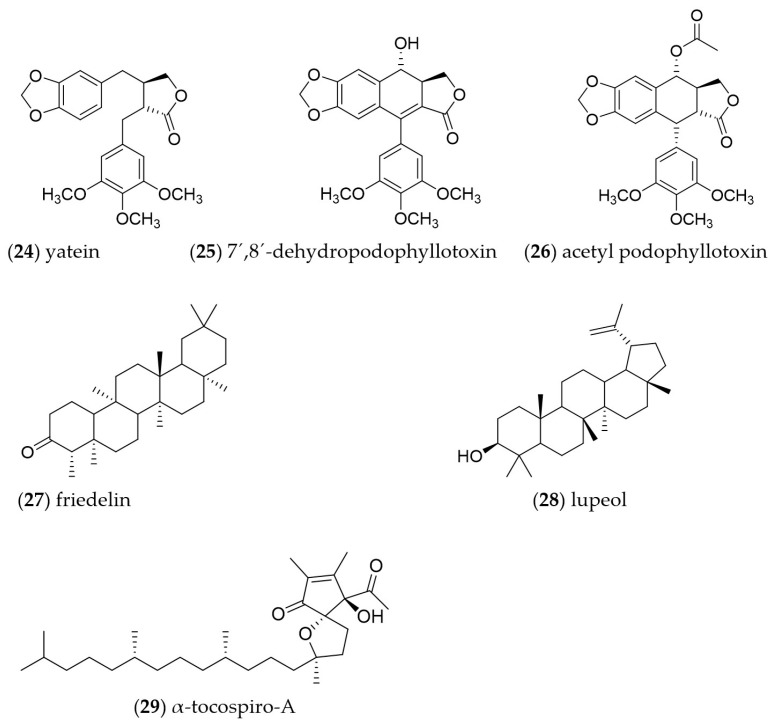
Chemical structures of compounds identified and isolated from the leaves of wild *B. fagaroides*.

Phytochemical analysis of the CH_2_Cl_2_ extract of the wild-type plant allowed the identification of three lignan-type compounds, namely, yatein (**24**), 7′,8′-dehydropodophyllotoxin (**25**) and acetyl podophyllotoxin (**26**), and two pentacyclic triterpenes, friedelin (**27**) and lupeol (**28**), as well as α-tocospiro-A (**29**) (Figure 6). Compounds **24**–**26** were isolated by column chromatography as described in Section 4.7.2 and identified by ^1^H and ^13^C NMR analysis and compared with data from the literature [5,6]. Compounds **27**–**29** were identified by gas chromatography coupled to mass spectrometry (GC-MS) (NIST 1.7a). The chromatogram is shown in Figure S1, and the identified metabolites are in Table S1 (Supporting Information). Unlike calluses, the wild plant produces many more volatile metabolites. Indeed, this study indicated that the leaves produced 21 metabolites distributed as six terpenes (14.9%), five fatty acids (7.4%), four alkanes (27.3%), one steroid (0.5%) and five triterpenes (22.0%). The most abundant metabolites were the alkane Hentriacontane with a retention time of 32.06 min and relative abundance of 19.1% and the triterpene Friedelin (RT = 40.28 min and relative abundance = 10.1%).

### 2.6. Evaluation of Cytotoxic Activity

The results of the cytotoxic evaluation of the CH_2_Cl_2_ extracts of the callus culture and wild plant are displayed in Table 2. It was generally observed that the wild-type plant extract showed important cytotoxicity against HepG2 (IC_50_ = 20.3 ± 3.38), HeLa (IC_50_ = 18.6 ± 5.2) and PC3 (IC_50_ = 12.6 ± 4.6), this last being the most sensitive. On the other hand, this extract did not show activity against H1299 (IC_50_ = 69.1 ± 6.2), and, when compared with its effect against the non-cancerous cell line HFF-1 (IC_50_ = 79.6 ± 5.2), selectivity for cancer cell lines was observed. Treatment of the five cell lines with the callus culture extract resulted in a negligible effect against all the cell lines tested: HepG2 (IC_50_ = 155.4 ± 4.95), HeLa (IC_50_ = 72 ± 5), PC3 (IC_50_ = 141 ± 5.8), H1299 (IC_50_ = 210.4 ± 5) and HFF-1 (IC_50_ = 95.4 ± 6.2). The HeLa cell line was the most sensitive (IC_50_ = 72 ± 5). The statistical analysis can be found in Figure S8.

## 3. Discussion

The accumulation of secondary metabolites in plants is low and slow because it is spatially and temporally regulated. Indeed, it occurs in specific cells, organs and tissues in specific phases of the plant life cycle under seasonal or stress conditions [27,28]. Therefore, an alternative to produce these metabolites of interest is the use of in vitro callus culture systems. Callus induction involves a process of dedifferentiation and constant cell division, which depends on the type of explant, culture medium, type, concentration and combination of plant growth regulators [29,30,31].

The leaves produced by the cultivation of cuttings of *B. fagaroides* were 90% free of pathogens, so this method was effective not only for obtaining plant material but also for the dedifferentiation of explants and obtention of calluses.

However, there are few studies on callus formation and development in the Burseraceae family because some species are arboreal and woody, with a high content of endophytic fungi, and some of them are endangered and highly recalcitrant, which leads to reduced germination [15,24,32]. In addition, the copales and cuajiotes of the *Bursera* genus present difficulties for germination and/or obtaining seedlings and calluses from seeds. These difficulties are because the fruit has relatively hard covers and passes through the digestive system of certain species of birds, where it softens and facilitates germination [33,34]. Another option is to use pregermination treatments such as hydrochloric acid immersion or mechanical scarification to obtain plants in vitro. The use of some auxins to obtain callus in the genus *Bursera* has been reported. Indeed, in an ex vitro cuttings culture of *B. fagaroides*, treatment with 1500 ppm of indole butyric acid (IBA) allowed the formation of 70–85% of callus after four months [32]. On the other hand, the application of 1.5 mgL^−1^ of indol butyric acid (IBA) over stem explants of *B. laxiflora* allowed the induction of 80% of callus after 30 days [35]. In another work, using the combination of naphthalene acetic acid (NAA, 3 mgL^−1^) and 6-benzylaminopurine (BAP, 0.5 mgL^−1^), 95% of callus induction was obtained from *B. linaloe* at 40 days of treatment [36]. Among the different types of auxins is 2,4-D, a synthetic auxin that causes rapid cell proliferation, formation and optimal callus growth. In addition to being the most widely used growth regulator in cereal tissue culture, it has been used for callus induction by initiating dedifferentiation from day 5 in cultures [37,38,39]. Some studies suggest that, when the concentration of 2,4-D is equal to or higher than 3 mgL^−1^, disorganized growth is observed, which favors callus formation, but, when increasing concentrations are used, they cause inhibition of cell division [37,38,40].

In this study, the best callus induction response was observed with concentrations of 4 mgL^−1^ of 2,4-D in combination with NAA and Zeatin at a ratio of 1:1 mgL^−1^ in B5 medium, producing a yellow light-colored callus which was friable without oxidation, had high percentages of cell viability and had circular and elongated morphologies in the different growth stages (Figure 2D,E). These results partially coincide with those reported by Souza-Pádua and collaborators [41], who showed two types of callus morphologies in *Coffea arabica*.

It should be noted that the combination of the PGR together with the used medium in this investigation favored the accumulation of biomass, allowing the characterization of the culture. The growth curve determined by the points analyzed, reported in fresh and dry biomass, presented a sigmoid curve with four distinct phases (adaptation, exponential, stationary and death) (Figure 3A). In the exponential phase, the kinetic parameters μ = 0.059 days^−1^ and td = 11.76 days, with a growth rate greater than 28 days, were obtained. This is the first report on the characterization of a callus culture of *Bursera fagaroides*. Mishra and Kumar [24] performed callus induction on *Commiphora wightii* (Burseraceae) using 5 mgL^−1^ of 2,4-D and 0.5 mgL^−1^ of BAP, obtaining 5.78 g of maximum fresh biomass. On the other hand, Al-Abdallat and collaborators [25] obtained 32.41 g of fresh biomass and 0.89 g of dry biomass in a callus culture of *Commiphora gileadensis* (Burseraceae) using high concentrations of auxin with respect to cytokinin. In our study, we obtained slightly lower concentrations of dry biomass (0.78 g). This is probably due to the combination and concentration of the PGR used. In addition, the results obtained were compared with studies of the same family (Burseraceae); however, species of the genus *Bursera* are highly recalcitrant and difficult to propagate, as previously described [12,13]. It is important to perform growth kinetics to decide whether the crop should be replanted and to determine in which stage the highest amount of the secondary metabolite of interest is produced.

With the aim of identifying the blue fluorescent compound observed by TLC (Figure 4A), a GC-MS analysis of the extract from callus biomass at different culture stages was performed. This analysis showed the presence of a major compound, further identified as scopoletin (7-hydroxy-6-methoxy coumarin), at 14 to 56 days. It is a simple coumarin derived from the C6-C3 carbon skeleton. It should be noted that, in the *B. fagaroides* callus culture, scopoletin had two points of production (Figure 4C), the first one being associated with the beginning of cell growth, with a maximum peak at day 21, and it decreased drastically thereafter. This may be because the culture medium was supplemented with 4 mgL^−1^ of 2,4-D. Previous studies reported that, when scopoletin plus 1 mgL^−1^ of 2,4-D was added endogenously to *Nicotiana tabaccum* callus cells, they absorbed scopoletin from the medium, and it was accumulated it in its glycoconjugate (scopolin), mainly in the vacuoles [42,43]. Likewise, some elicitors have been used for its synthesis. For example, it has been reported that the synthesis of scopoletin was associated with cell growth in callus and suspension cultures of *Tilia americana* after the copper concentration was increased up to 1.2 μM [44], and copper sulfate stimulated its production in suspension cultures of *Angelica archangelica* [45]. The second point of scopoletin production started on day 42 (Figure 4C), with a peak on day 56, i.e., it was associated with the lack of nutrients in the culture medium. It should be noted that this compound is one of the phytoalexins induced by different types of stress [44,45,46].

There are very few reports on the effect of natural coumarins on cell division and callogenesis in plant tissues cultured in vitro [47]. The culture of *Ammi majus* callus on Linsmaier–Skoog’s medium with the PGRs NAA and BAP (1:1 mgL^−1^) was the best for the accumulation of coumarins (furan coumarins) that favor the formation of embryogenic callus. The levels of these metabolites in the in vitro culture were several times higher than those found in the vegetative organs of the plants [48].

It should be noted that this metabolite has been found to possess antibacterial, cytotoxic and antifungal activities [49,50,51,52] and prevent weight gain and lipemia effect in mice [53]. It has been isolated from several plant families such as Apiaceae, Rutaceae, Asteraceae and Fabaceae, to mention a few [54]. Also, it has also been found in the bark of some species of the genus *Bursera* such as *B. grandifolia* [53], *B. serrata* [51,52] and *B. morelensis* [55], as well as in *B. simuraba* leaves [56]. However, so far, there are no reports of its presence in the leaves of *B. fagaroides*.

Phytochemical investigation of the dichloromethane extract of *B. fagaroides* callus biomass demonstrated the presence of phytosterols in addition to scopoletin. The mixture of γ-sitosterol and stigmasterol was isolated from the extract after chromatographic separation, and their structures (Figure 5 and Figure 6) were identified by comparing their spectroscopic data with those reported [55,56,57]. Also, we compared their mass spectra with the NIST database version 1.7. These compounds are present in a variety of plant species; their biological activities have been thoroughly investigated, and their pharmaceutical effects have been established. These compounds also showed anti-inflammatory, antibacterial, and antitumor effects. In general, approximately 250 phytosterols are found in plants, including sitosterol, stigmasterol, campesterol, brassicasterol, ergosterol and β-sitosterol. It has been proposed that a phytosterol-rich diet may reduce cancer risk by 20% [58]. The C-24 alkyl plant sterols are present in all higher plants, and these are found in the lipid-rich fractions of plants [59]. A number of in vitro and in vivo studies have demonstrated the cytotoxic and antitumoral activity of sterols, mainly β-sitosterol, an isomer of γ-sitosterol found in *B. fagaroides* callus biomass [60,61].

**Figure 7:**
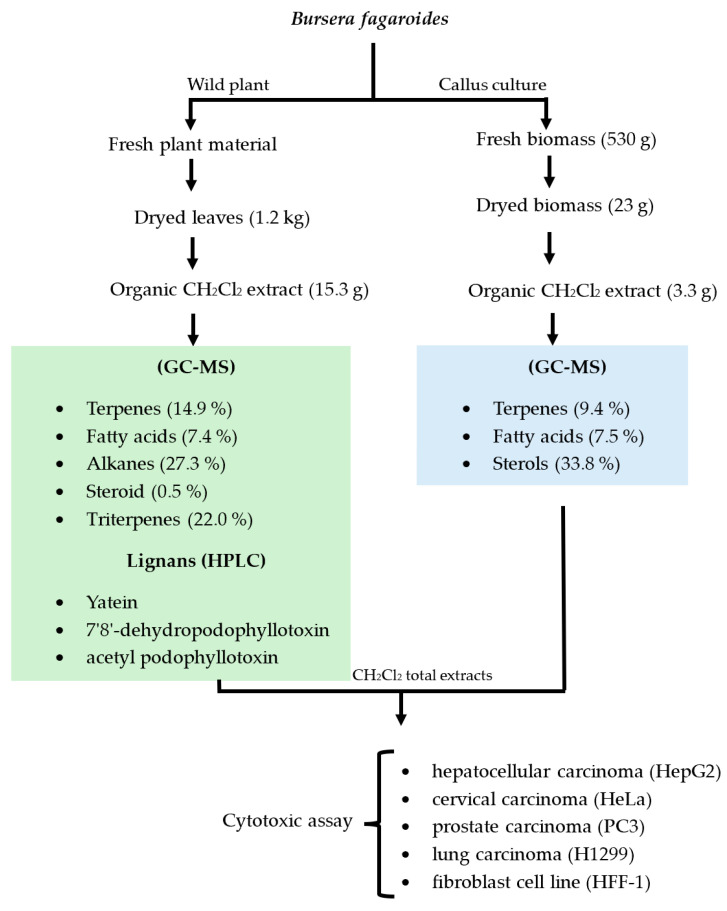
Extraction process and cytotoxic evaluation for leaves and callus culture of *Bursera fagaroides*.

On the other hand, the phytochemical study of the dichloromethane extract from leaves demonstrated the presence of lignans and triterpenes principally (Figure 7). These results are in accordance with our previous studies on the bark of *B. fagaroides*, where we described the isolation of several aryltetralin-type lignans with potent cytotoxic activity against prostate cancer cells (PC3) compared to other human cancer cells [7]. In this case, the lignans obtained in this work (yatein, 7′8′-dehydropodophyllotoxin and acetyl podophyllotoxin) were previously obtained from the bark.

The difference in the metabolic profile of callus biomass compared to wild plant leaves may be due to the age of the plant and/or nutritional composition of the B5 medium, which contains all the micronutrients and macronutrients, vitamins, organic supplements and plant growth regulators necessary for the growth and proliferation of plant cells in vitro.

Results of the evaluation of the cytotoxic activity established that the leaf extract was more cytotoxic than the callus extract (Table 2). The wild plant showed the highest activity against the PC3 cell line (IC_50_ = 12.6 ± 4.6), while the callus biomass extract was more toxic against the HeLa cell line (IC_50_ = 72 ± 5). This difference could be due to the presence of the cytotoxic lignans yatein (**24**), 7′,8′-dehydropodophyllotoxin (**25**) and acetyl podophyllotoxin (**26**) in the leaves, which showed IC_50_ values of and 2.42 × 10^−5^ and 0.0109 µM when probed against PC-3 cells [7]. Nevertheless, callus biomass does not produce lignans; instead, it synthesizes the coumarin scopoletin, which has been described as possessing cytotoxic activity against the human cervical cancer cell line HeLa (IC_50_ = 7.5 to 25 µM) [62].

It is important to underline the benefits of using biotechnological platforms, which, when applied properly, can lead to constant production of biomolecules, in contrast to wild plant material, which is subjected to weather, growth, biotic and abiotic factors and other ecosystem conditions.

In the future, it is important to start liquid suspension cultures that can be scaled up into bioreactors.

## 4. Materials and Methods

### 4.1. General

Compounds were isolated by means of open column chromatography (CC). Analytical TLC was carried out on precoated silica gel (TLC, ALUGRAM^®^ SIL G/UV 254 silica gel plates 60 F_254_ plates). The isolation procedures and purity of compounds were checked by thin-layer chromatography (TLC), visualized by means of UV light and sprayed with Ce(SO_4_)_2_ 2(NH_4_)2SO_4_ 2H_2_O. All ^1^H and ^13^C NMR experiments were recorded on a Bruker AVANCE III HD 500 MHz at 500 and 125 MHz, respectively, using CDCl_3_ with tetramethylsilane (TMS) as internal standard. The dichloromethane extracts of the callus and wild-type leaves were analyzed by GC-MS Agilent GC 6890, MSD 5973N (Agilent Technologies, USA), to determine the chemical composition of the major compounds. The analysis was performed using the HP-5MS column (30 mm × 0.25 mm × 0.25 µm). The carrier gas was helium with a gas flow rate of 1 mLmin^−1^ and a linear velocity of 37 cm/s. The injector temperature was set to 250 °C (splitless). The initial oven temperature was 40 °C and was increased to 250 °C for 5 min and by 10 °C/min, and the final temperature was maintained at 285 °C for 20 min. The mass spectrometer was operated in the electron ionization mode at 70 eV, and the electron multiplier voltage was 1859 V. Compounds were identified by comparison of retention times and fragmentation patterns of reference compounds from the NIST database version 1.7a. Paclitaxel, Fluorescein Diacetate, scopoletin, Polyvinylpyrrolidone (PVP), phytagel, 2,4-D (2,4-dichlorophenoxyacetic Acid), NAA (Naphthaleneacetic Acid), Zeatin, dimethyl sulfoxide (DMSO) and RPMI-1640 medium were purchased from Sigma Aldrich (México City, México). Curamycin antibiotic was from Agricola 500 (México). Gentamicin was from AMSA. HepG2 (hepatocellular carcinoma), HeLa (cervical carcinoma), PC3 (prostate carcinoma) and H1299 (lung carcinoma) cells were from ATCC (American Type Culture Collection, USA). DMEM and fetal bovine serum (SFB) were from Invitrogen (Thermo Fisher Scientific, Waltham, MA, USA). CellTiter 96^®^ AQueous One Solution Cell Proliferation Assay kit was from Promega (Madison, WI, USA).

### 4.2. Identification of Plant Material

A specimen of *Bursera fagaroides* (Kunth) Engl. was collected on July 2021 in the locality “El Mango” in the municipality of Puente de Ixtla (14 Q 046300, UTM 2043732, DDQH9), altitude: 1382 m, by Biologist Fidel Ocampo Bautista and was identified by Biologist Gabriel Flores Franco. It was deposited in the HUMU Herbarium of the Universidad Autónoma del Estado de Morelos (UAEM) with voucher number 39796.

### 4.3. Obtaining Plant Material

Thirty fragments of approximately 20 cm in length were randomly cut from terminal branches of an adult tree and submerged in water for 12 h. Subsequently, a rooting agent (Raizone Plus) was added to each tip, and 10 of them were placed per pot, constituting a total of 3 pots for the study, which contained coconut fiber as substrate (n = 10). The experiments were conducted in triplicate. The pots were placed in greenhouse conditions at 41 ± 2° C and watered every third day by misting for 139 days. The plant material obtained was used for the establishment of the in vitro culture.

### 4.4. Callus Induction

Young leaves of between 2 and 3 cm in length developed from the stake culture were used as explants for callus induction. They were washed with sterile miliQ water and neutral soap (Hycel) for 4 min, then an antifungal was added, followed by successive washes with 40% and 10% sodium hypochlorite for 3 min each. Subsequently, inside the laminar flow hood, they were immersed with a broad-spectrum antibiotic (Curamycin Agricola 500) for 4 min, rinsed with sterile miliQ water and dried with filter paper. Finally, cuts were made with a scalpel, and they were placed in 9 cm diameter Petri dishes containing 25 mL of Murashige and Skoog (1962) culture medium using as carbon source 30 gL^−1^ of sucrose and supplemented with 6 gL^−1^ of Polyvinylpyrrolidone (PVP), 10 mgL^−1^ of gentamicin, 1 and 5 gL^−1^ of phytagel, adjusting the pH to 5.7 plus phytoregulators. The culture medium was previously sterilized at 121 °C, 15 psi, for 15 min. Ten explants were seeded per Petri dish with three replicates and maintained for a 16/8 light–dark photoperiod at 25 ± 2 °C for 30 days. To determine which combination induced better callus formation, ten combinations with MS medium plus B5 medium (Gamborg, 1968) and ten combinations with 2,4-D plus Kinetin (2,0; 2,1; 2,2; 3,1; 3,2; 3,3; 4,1; 4,2; 4,3 and 4,4 mgL^−1^) were used. Callus obtained from the 2,4-D:Kinetin combination (2:2 mgL^−1^), which showed better appearance, was transferred to 20 mL B5 medium containing 4 mgL^−1^ of 2,4-D, 1 mgL^−1^ of NAA and 1 mgL^−1^ of Zeatin using as carbon source sucrose (30 gL^−1^) and supplemented with PVP (6 gL^−1^) and phytagel (1. 5 gL^−1^) and adjusting the pH to 5.7. Friable calluses were obtained and reseeded every 28 days for 6 months. The cultures were incubated at 25 ± 2 °C in a photoperiod with white fluorescent light (50 µmol m^−2^s^−1^).

### 4.5. Growth Kinetics of Callus Culture

Growth kinetics tests were performed in triplicate by placing an inoculum with 1 g of fresh biomass per flask with the previously mentioned culture conditions and incubating it for a photoperiod of 16 h light/8 h dark at 25 ± 2 °C. Fresh and dry biomass was determined at seven-day intervals after inoculation up to 63 days. During the assay, the weight of fresh biomass was recorded by placing it on previously weighed filter paper. Subsequently, to determine the dry biomass, the plant material was lyophilized at −50 °C and 0.1–0.3 mBar until a constant weight was reached. The callus growth curve was plotted with the culture time against the weights of the fresh and dry biomasses to show the phases of cell growth (adaptation, exponential, stationary and death). Kinetic parameters were calculated: the specific growth rate µ (time^−1^), the cell doubling time (td) using the following formula td = ln(2)/μ [15,16,17,18,19,20,21,22,23] and the growth index (GI) given by the final dry biomass minus the initial dry biomass divided by the initial dry biomass.

### 4.6. Cell Morphology and Viability

Cell viability was measured with the fluorescein diacetate method (FAD) [63]. The percentage viability of each sample was determined based on the number of cells that were stained green divided by the total number of cells counted. For both cell morphology and viability, observations were made using an epifluorescence microscope [64]. Objectives with 40× and 10× magnification were used.

### 4.7. Phytochemical Analysis of the Callus Culture

#### 4.7.1. Obtaining the Extracts

The dry plant biomass of the callus collected from the different growth stages was used to determine the production of scopoletin with respect to time of growth. For this purpose, the samples were ground to a fine powder. The pulverized material was macerated in triplicate with dichloromethane (CH_2_Cl_2_) for 24 h with a ratio of 1 g of plant material per 10 mL of solvent. The samples were concentrated by separating the solvent by distillation at reduced pressure using a rotary evaporator (Büchi R-100, BÜCHI Labortechnik AG, Flawil, Swiss) [5,6]. The product obtained was brought to complete dryness at room temperature, and the procedure was repeated three times. The yield expressed in percentage was determined for each of the extracts and stored until further use.

To separate, obtain and identify the majority compounds, a sample of 530 g of fresh callus biomass from 15 to 35 days of culture was lyophilized [65], obtaining 23 g of dry biomass which was crushed until pulverized, then macerated with CH_2_Cl_2_ following the procedure previously described, obtaining 3.3 g of extract (0.6% yield).

#### 4.7.2. Chemical Profile and Identification of Major Compounds

Once the extracts from each day of callus culture were obtained, the chemical profile was analyzed.

First, thin-layer chromatography was performed using hexane–ethyl acetate (5:5) as the elution system. The plates without developer were exposed to irradiation with shortwave UV light at 254 nm and longwave UV light at 360 nm and then ammoniated with ammoniated ceric sulfate as a chemical developer. The plates without chemical treatment and exposed to irradiation with ultraviolet light at 360 nm allowed the identification of scopoletin (bright blue spot with Rf = 0.32) as the major compound.

Subsequently, to confirm the presence of scopoletin in the callus culture, a 10 µL aliquot of the extract was injected with CH_2_Cl_2_ from the culture at day 49 (2 mgmL^−1^) using a high-performance liquid chromatograph (Waters 996, Waters Corporation, Milford, MA, USA) with binary pumps (2695) coupled to a diode array detector (2996) with a UV detection range from 190 to 600 nm and operated by Millenium System Manager Software (Empower 1) (version 4.0) [49] using an elution system of water/acetonitrile mixture (70:30) and maintaining a constant flow rate of 1 mLmin^−1^ for 30 min. The column used was a Supelco RP-18 (5 μm, 4.6 mm × 25 cm).

For the quantification of scopoletin, a calibration curve was calculated. A commercial standard was used as reference. Five different concentrations were used: 50, 25, 12.5, 6.25 and 3.125 µgmL^−1^ of the compound dissolved in HPLC-grade acetonitrile. The chromatogram was read at a wavelength of 350 nm.

Finally, the production of scopoletin was quantified for each point of the growth kinetics by plotting the dry biomass against the production of this metabolite. The analysis was performed in triplicate, and the results are shown as µgmg^−1^ dry biomass. Finally, the results obtained were compared against the extract of the leaves of the wild plant.

After performing the identification and quantification analysis of scopoletin on the different days of callus culture, we proceeded to separate and identify the majority compounds. The extract with CH_2_Cl_2_ (3.3 g) obtained from the callus culture from days 15 to 35 was fractionated by open column chromatography, previously packed with silica gel (6 g, 70–230 mesh; Merck KGaA, Darmstadt, Germany). Fractions of 150 mL were collected, obtaining 182 fractions monitored by TLC. The fractions that showed similarity in TLC were grouped, obtaining 6 groups: NPM 1 (1–25) eluted with hexane–acetate (95:5), NPM 2 (26) eluted with hexane–acetate (90:10), NPM 3 (27–53) eluted with hexane–acetate (85:15), NPM 4 (107–114), NPM 5 (115–151) eluted with hexane–acetate (75:25) and NPM 6 (152–182) eluted with hexane–acetate (70:30).

The NPM 2 group (white crystals, 12 mg) was shown by TLC to contain a single compound, and the GC/MS analysis indicated that it was constituted by gamma sitosterol (**20**). The NPM 4 group (light yellow wax, 4.9 mg) presented a mixture of the compounds dehydrodiosgenin (**21**) and stigmaesterol (**22**). Finally, the NPM 6 group (light yellow solid, 28.1 mg) indicated the presence of a mixture of several compounds; the main one was scopoletin (**23**).

### 4.8. Phytochemical Analysis of Wild Plant Leaves

#### 4.8.1. Obtaining the Extracts

To obtain the extracts from the wild plant, fresh leaves were first collected and dried in the open air and under shade for 20 days, obtaining 1.2 kg of dry plant material. Subsequently, it was ground to a particle size of 2–5 mm, and maceration was performed in triplicate with CH_2_Cl_2_, following the same protocol. A 15.3 g extract (1.25% yield) was obtained.

#### 4.8.2. Chemical Profile and Identification of Major Compounds

The isolation and purification of compounds from the CH_2_Cl_2_ extracts were carried out by successive fractionation by column chromatography using various organics solvents. From the last open column fractionation on silica gel 60 (15 g, 70–230 mesh; Merck) eluted with hexane–ethyl acetate, five groups were obtained. From these fractions, NPM-40-2, NPM-40-3 and NPM-40-4 were passed through activated carbon to remove the color.

The final separation of the compounds was performed by HPLC analysis, as reported by Rojas-Sepúlveda in 2012 [5], because the three samples were found to contain a mixture of several compounds. For this purpose, a semi-preparative X-Terra RP-18 reversed-phase column (7.8 mm × 50 mm; 5 µm particle size) was used; the mobile phase was 25:75 acetonitrile–water at a flow rate of 1.0 mLmin^−1^ with a separation module and a diode array detector with detection at 215, 250, 300 and 350 nm and a flow rate of 1 mLmin^−1^ with an isocratic profile for 30 min. Each injection was 10 μL, and the solvents used were HPLC grade.

Fraction NPM-40-2 was purified by HPLC to afford 2.9 mg of a compound with tR = 13.57 and identified as yatein (**24**, tR = 13.57 min). Similarly, the purification of fraction NPM-40-3 allowed 1.6 mg of 7′,8′-dehydropodophyllophyllotoxin to be obtained (**25**, tR = 11.76 min). Finally, HPLC fractionation of NPM-40-4 afforded 1.5 mg of acetyl podophyllotoxin (**26**, tR = 16.24 min). It should be noted that, in addition to the fractionation, the volatile compounds from the total extract were separated and analyzed by GC/MS, showing friedelin (**27**), lupeol (**28**) and α-tocospiro-A (**29**) as the main compounds.

### 4.9. Cytotoxicity Assay

The cytotoxic activity of the CH_2_Cl_2_ extracts from the leaves of the wild plant and the callus culture was evaluated using the MTS method [66]. Different human cancer cell lines were used such as HepG2 (hepatocellular carcinoma), HeLa (cervical carcinoma), PC3 (prostate carcinoma) and H1299 (lung carcinoma). The cell lines were obtained from ATCC. We also included an immortalized human fibroblast cell line (HFF-1) as a control of non-cancerous cells. The PC3 and H1299 cell lines were cultured in RPMI-1640 medium, while HepG2, HeLa, MCF7 and HFF-1 were cultured in DMEM supplemented with 10% fetal bovine serum and 2 mM glutamine. All cultures were incubated at 37 °C in a 5% CO_2_ atmosphere.

Cells (5 × 10^3^ cells/well) were seeded in 96-well plates to initiate cytotoxic evaluation. The stock solutions of each extract were prepared from 10 mgmL^−1^ (10,000 µgmL^−1^). A 1 mg amount of extract was weighed and dissolved in 100 µL of DMSO, and a concentration/response curve was generated using concentrations 200, 100, 50, 25 and 12.5 µgmL^−1^, while, in HFF-1 cells, the concentrations were 400, 200, 100, 50 and 25 µgmL^−1^. The negative control was 0.5% DMSO in a culture medium. After treatment, the cells were incubated at 37 °C in 5% CO_2_ atmosphere. Paclitaxel was used as a positive control. For determining the number of viable cells in proliferation, we used a CellTiter 96^®^ AQueous One Solution Cell Proliferation Assay kit (Promega Corporation. Madison, WI, USA), following the manufacturer’s instructions. Cell viability was determined by absorbance at 450 nm using an automated ELISA reader (Promega, Madison, WI, USA). The experiments were conducted in triplicate in three independent experiments. Data were analyzed using the Prism 8.0 statistical program (Graphpad Software Inc., La Jolla, CA, USA), and the half-maximal inhibitory concentrations (IC_50_) were determined by regression analysis.

### 4.10. Statistical Analysis

All experiments were conducted with three replicates for each treatment and were repeated three times and are presented as means ± SDs. Statistical analyses were performed by one-way analysis of variance (ANOVA), followed by a multiple comparison of means (Tukey). GraphPad Prism 9.4.1 (681) software was used, with a *p* value ˂ 0.05 being considered to indicate statistical significance.

## 5. Conclusions

For the first time, conditions were established to obtain an in vitro culture of friable callus from *B. fagaroides* using a combination of PGRs: 4 mgL^−1^ 2,4-D, 1 mgL^−1^ NAA and 1 mgL^−1^ Zeatin. Four phases of cell growth were clearly identified in this culture. The maximum cell growth point was identified at day 28 with a μ = 0.059 days^−1^ and td = 11.8 days. It was observed that, in this culture, scopoletin was synthesized as the main compound (inducible as a phytoalexin by the addition of high concentrations of 2,4-D, as well as by the absence of nutrients in the culture medium), as well as γ-sitosterol, dehydrodiosgenin and stigmasterol.

Open column chromatography was used to separate and identify three abundant compounds, yatein, acetyl podophyllotoxin and 7′,8′-dehydropodophyllotoxin, in the extract of the leaves of the wild plant. It should be noted that this is the first time yatein has been described in leaves.

The cytotoxic activity of four cell lines was tested, the most sensitive being PC3 (IC_50_ of 12.6 ± 4.6) in the wild-type plant extract and HeLa (IC_50_ of 72 ± 5) in the callus culture extract.

From the friable callus obtained in this study, it is now possible to establish suspension cultures that not only provide the metabolites of interest, but also allow their production to be increased through bioreactor scale-up.

## Figures and Tables

**Table 1:** Effect of plant growth regulators and culture media on callus induction of *Bursera fagaroides* leaf explants.

MEDIA	PGR (mgL^−1^)		CALLUS (%) ± DE
	2,4-D	KINETIN	
MS	2	0	0.00 ± 0.00 ^d^
MS	2	1	0.00 ± 0.00 ^d^
MS	2	2	86.67 ± 5.77 ^a^
MS	3	1	0.00 ± 0.00 ^d^
MS	3	2	0.00 ± 0.00 ^d^
MS	3	3	0.00 ± 0.00 ^d^
MS	4	1	0.00 ± 0.00 ^d^
MS	4	2	33.33 ± 5.77 ^b^
MS	4	3	0.00 ± 0.00 ^d^
MS	4	4	26.67 ± 5.77 ^b^
B5	2	0	0.00 ± 0.00 ^d^
B5	2	1	13.33 ± 5.77 ^c^
B5	2	2	16.67 ± 5.77 ^c^
B5	3	1	0.00 ± 0.00 ^d^
B5	3	2	0.00 ± 0.00 ^d^
B5	3	3	0.00 ± 0.00 ^d^
B5	4	1	0.00 ± 0.00 ^d^
B5	4	2	0.00 ± 0.00 ^d^
B5	4	3	0.00 ± 0.00 ^d^
B5	4	4	0.00 ± 0.00 ^d^

PGR = plant growth regulators. Data are the mean ± standard deviation. Values with the same letters in the columns are not statistically different according to the multiple comparison of means test (Tukey), which defined significance as *p* ˂ 0.05.

**Table 2:** IC_50_ values (µgmL^−1^) of CH_2_Cl_2_ extracts of wild-type plant and callus culture of *B. fagaroides*.

Extract	HepG2	HeLa	PC3	H1299	HFF-1
Leaves ^b^	20.3 ± 3.38 ****	18.6 ± 5.2 ***	12.6 ± 4.6 ****	69.1 ± 6.2 ****	79.6 ± 5.2 *
Callus ^b^	155.4 ± 4.95 ****	72 ± 5 ***	141 ± 5.8 ****	210.4 ± 5 ****	95.4 ± 6.2 *
Paclitaxel ^a^	10.12 ± 2.5	40.23 ± 7.2	17.5 ± 2.8	45.25 ± 7	989 ± 21

^a^ Paclitaxel values are in nM. ^b^ The extracts of leaves and callus are in µgmL^−1^. Values are expressed as the mean ± SD of three independent experiments. Student’s *t*-test analysis showed that all the IC50 values between leaves and callus were statistically significant with the following *p* values: * *p* = 0.0276; *** *p* = 0.0002; **** *p* ˂ 0.0001.

## Data Availability

Data are contained within the article and Supplementary Materials.

## Supplementary Materials

The following supporting information can be downloaded at: https://www.mdpi.com/article/10.3390/plants13121622/s1, Table S1: Compounds found in dichloromethane extracts of wild plant leaves and callus culture of *Bursera fagaroides*. Figure S1: GC-MS general chromatogram of the CH_2_Cl_2_ extract of wild plant leaves. Figure S2. GC-MS general chromatogram of the CH_2_Cl_2_ extract of the in vitro callus culture. Figure S3. GC-MS chromatogram of NPM 2 group corresponding to γ-sitosterol (**20**), RT = 35.11 min. Figure S4. GC-MS chromatogram of NPM 4 group corresponding to dehydrodiosgenin (**21**), RT = 31.55 min, and stigmasterol (**22**), RT = 35.24 min. Figure S5. GC-MS chromatogram of NPM 6 group corresponding to scopoletin (**23**), RT = 19.26 min. Figure S6. HPLC chromatogram of the NPM-40-2 fraction containing the compound yatein (**24**), tR = 13.57 min, 250 nm. Figure S7. HPLC chromatogram of the NPM-40-3 and NPM-40-4 fractions containing the compounds 7′,8-dehydropodophyllotoxin (25), tR = 11.76 min, and acetyl podophyllotoxin (**26**), tR = 16.24 min, 250 nm.
