# Supplementary material for: Phytochemical Profiles and Cytotoxic Activity of Bursera fagaroides (Kunth) Engl. Leaves and Its Callus Culture
Source: Plants (Basel). 2024 Jun 12;13(12):1622. doi: 10.3390/plants13121622 (PMC11207444; doi:10.3390/plants13121622)

Supporting Information

Phytochemical profiles and cytotoxic activity of *Bursera fagaroides* (Kunth) Engl.) leaves and its callus culture

Nancy Pérez-Mejía[1], María Luisa Villarreal[1], Jessica Nayelli Sánchez-Carranza[2], Leticia González-Maya[2], Manasés González-Cortazar[3], Anabel Ortíz-Caltempa[1]*and Laura Alvarez[4]*

1 Centro de Investigación en Biotecnología, Universidad Autónoma del Estado de Morelos, Avenida Universidad 1001, Col. Chamilpa, Cuernavaca C. P. 62209, México; maria.perezme@uaem.edu.mx (N.P.-M.); luisav@uaem.mx (M.L.V.)

2 Facultad de Farmacia, Universidad Autónoma del Estado de Morelos, Avenida Universidad 1001, Col. Chamilpa, Cuernavaca C. P. 62209, México. jessica.sanchez@uaem.mx (J.N.S.-C.); letymaya@uaem.mx (L.G.-M.)

3 Centro de Investigación Biomédica del Sur, IMSS, Calle República Argentina No. 1, Col. Centro, Xochitepec C. P. 62790, México; gmanases@hotmail.com

4 Centro de Investigaciones Químicas, Universidad Autónoma del Estado de Morelos, Avenida Universidad 1001, Col. Chamilpa, Cuernavaca C.P. 62209, México; lalvarez@uaem.mx

* Correspondence: anabel@uaem.mx (A.O.-C.); lalvarez@uaem.mx (L.A.)

Content:

Table S1. Volatile compounds found in the dichloromethane extracts of wild plant leaves and callus culture of *Bursera fagaroides*.

Figure S1. GC-MS General chromatogram of the CH_2Cl_2 extract of wild plant leaves.

Figure S2. GC-MS General chromatogram of the CH_2Cl_2 extract of the *in vitro* callus culture.

Figure S3. GC-MS Chromatogram of NPM 2 group corresponding to γ-sitosterol (**20**) RT=35.11 minutes.

Figure S4. GC-MS Chromatogram of NPM 4 group corresponding to dehydrodiosgenin (**21**) RT=31.55 minutes and stigmasterol (**22**) RT=35.24 minutes.

Figure S5. GC-MS Chromatogram of NPM 6 group corresponding to scopoletin (**23**) RT=19.26 minutes.

Figure S6. HPLC chromatogram of the NPM-40-2 fraction containing the compound yatein (**24**) RT = 13.57 minutes. 250 nm.

Figure S7. HPLC chromatogram of the NPM-40-3 and NPM-40-4 fractions containing the compounds 7′8-dehydropodophyllotoxin (**25**) RT= 11.76 minutes and acetyl podophyllotoxin (**26**) tR= 16.24 minutes, 250 nm.

Table S1: Volatile compounds found in the dichloromethane extracts of wild plant leaves and callus culture of *Bursera fagaroides*.

Wild plant leaves extract						Callus culture				
**No**	**Compounds**	**RT (min)**	**Relative Content (%)**	**Molecular Formula**	**Molecular Weight**	**Compounds**	**RT (min)**	**Relative Content (%)**	**Molecular Formula**	**Molecular Weight**
1	Hexahydrofarnesyl acetone	17.67	4.4	$C_{18}H_{36}O$	268.3	2,5-Octadecadiynoic acid, methyl ester	15.86	1.7	$C_{19}H_{30}O_2$	290.23
2	2-Hydroxy-1,1,10-trimethyl-6,9-epidioxydecalin	20.20	0.6	$C_{13}H_{22}O_3$	226.2	Geranyl isovalerate	16.69	1.0	$C_{15}H_{26}O_2$	238.19
3	1-Hexadecyn-3-ol, 3,7,11,15-tetramethyl-	20.58	0.5	$C_{20}H_{38}O$	294.3	Hexahydrofarnesyl acetone	17.56	0.5	$C_{18}H_{36}O$	268.28
4	4,8,12,16-Tetramethylheptadecan-4-olide	22.45	1.4	$C_{21}H_{40}O_2$	324.3	Palmitic acid	18.36	2.8	$C_{17}H_{34}O_2$	270.26
5	Methyl tetracosanoate	27.28	1.4	$C_{25}H_{50}O_2$	382.4	7-Methyl-Z-tetradecen-1-ol acetate	23.58	3.0	$C_{17}H_{32}O_2$	268.24
6	α-tocospiro A	29.50	8.1	$C_{29}H_{50}O_4$	462.4	2,6,10,14,18-Pentamethyl-2,6,10,14,18-eicosapentaene	28.49	2.0	$C_{25}H_{42}$	342.33
7	Nonacosane	29.69	2.4	$C_{29}H_{6}0$	408.5	7-Dehydrodiosgenin	31.55	1.2	$C_{27}H_{40}O_3$	412.30
8	Methyl hexacosanoate	30.10	2.4	$C_{27}H_{54}O_2$	410.4	Gamma sitosterol	35.11	32.0	$C_{29}H_{50}O$	412.39
9	Pentacosanoic acid, 2,10-dimethyl-, methyl ester	30.80	0.9	$C_{28}H_{56}O_2$	424.4	estigmasterol	35.24	0.6	$C_{29}H_{50}O$	414.39
10	Ethyl cholate	31.83	0.5	$C_{26}H_{44}O_5$	436.3	Betulinaldehyde	37.83	5.9	$C_{30}H_{48}O_2$	440.37
11	Hentriacontane	32.06	19.1	$C_{31}H_{64}$	436.5					
12	methyl octacosanoate	32.49	1.3	$C_{29}H_{58}O_2$	438.4					
13	Vitamin E	32.62	0.5	$C_{29}H_{50}O_2$	430.4					
14	Octadecane, 3-ethyl-5-(2-ethylbutyl)-	33.38	0.8	$C_{26}H_{54}$	366.4					
15	Triacontane-1,30-diol	34.17	5.0	$C_{30}H_{62}O_2$	454.5					
16	Azafrin	35.72	0.8	$C_{27}H_{38}O_4$	426.3					
17	Betulin	38.78	2.8	$C_{30}H_{50}O_2$	442.4					
18	Beta-Amyrin	36.19	1.5	$C_{30}H_{50}O$	426.4					
19	Betulinaldehyde	36.31	0.8	$C_{30}H_{48}O_2$	440.4					
21	Lupen-3-one	36.59	1.3	$C_{30}H_{48}O$	424.4					
22	Lupeol	37.12	6.3	$C_{30}H_{50}O$	426.4					
22	Friedelin	40.28	10.1	$C_{30}H_{50}O$	426.4					

Figure S1: GC-MS General chromatogram of the CH_2Cl_2 extract of wild plant leaves.

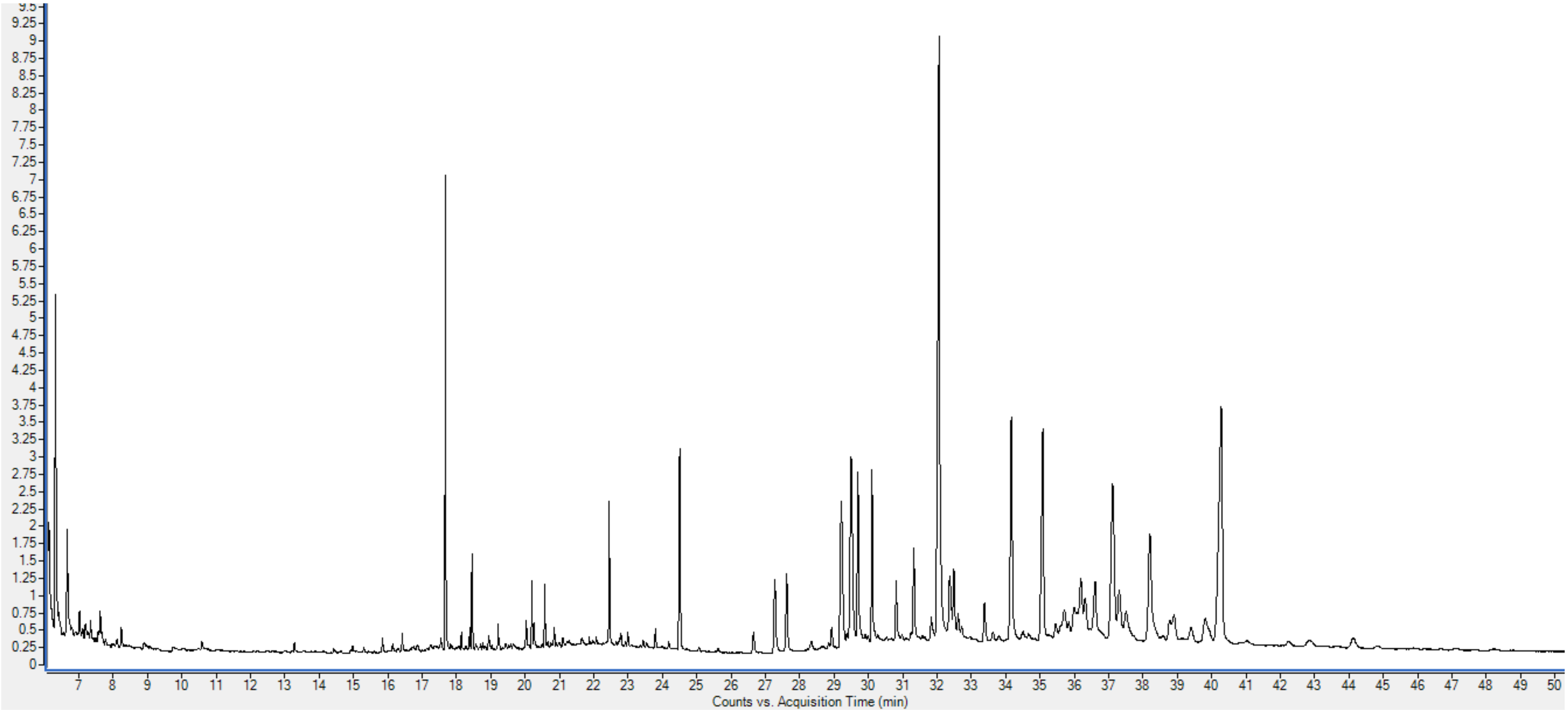

Figure S2. GC-MS General chromatogram of the CH_2Cl_2 extract of the *in vitro* callus culture.

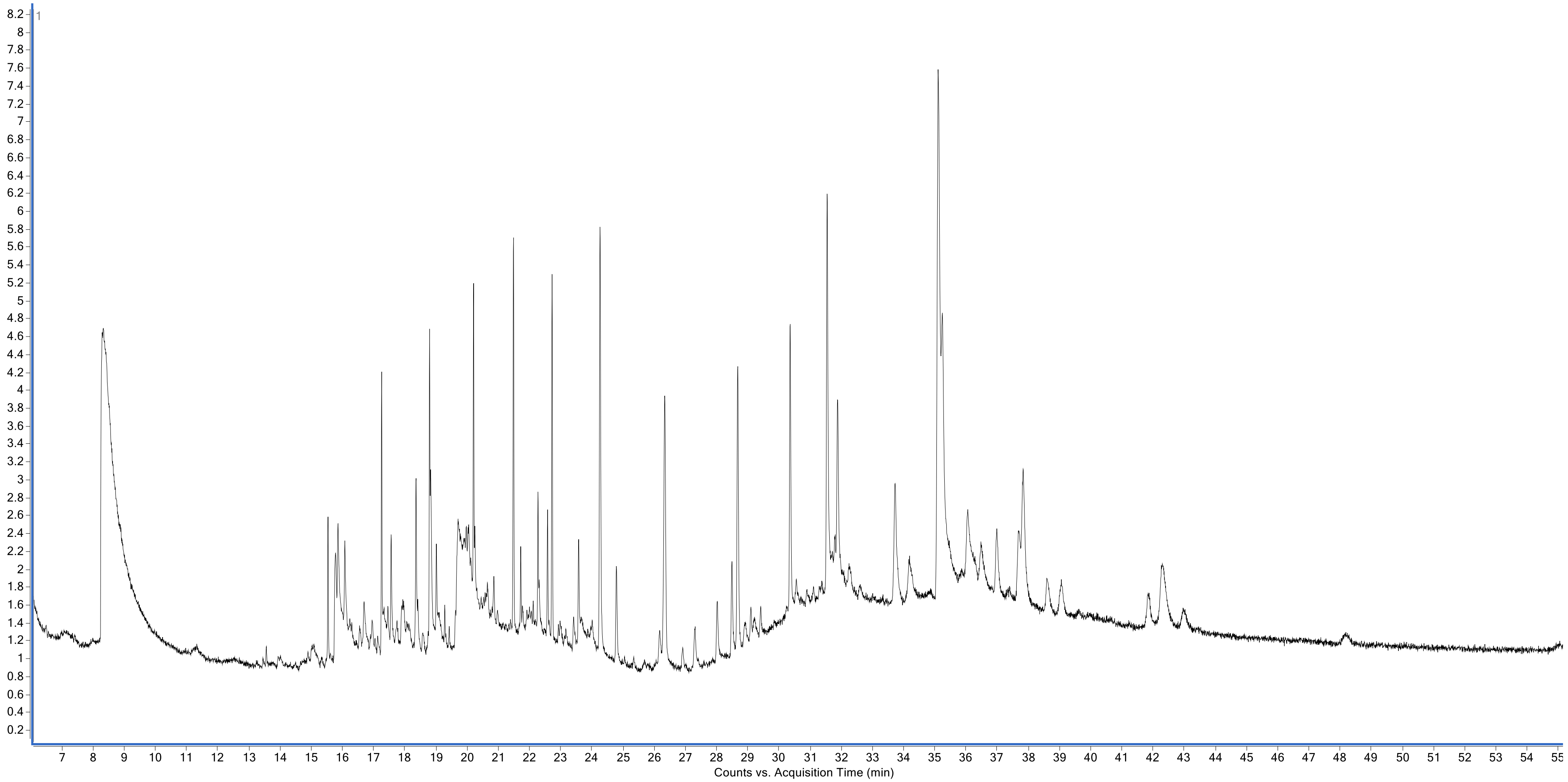

Figure S3. GC-MS Chromatogram of NPM 2 group corresponding to γ-sitosterol (**20**) RT=35.11 minutes.

(**20**)

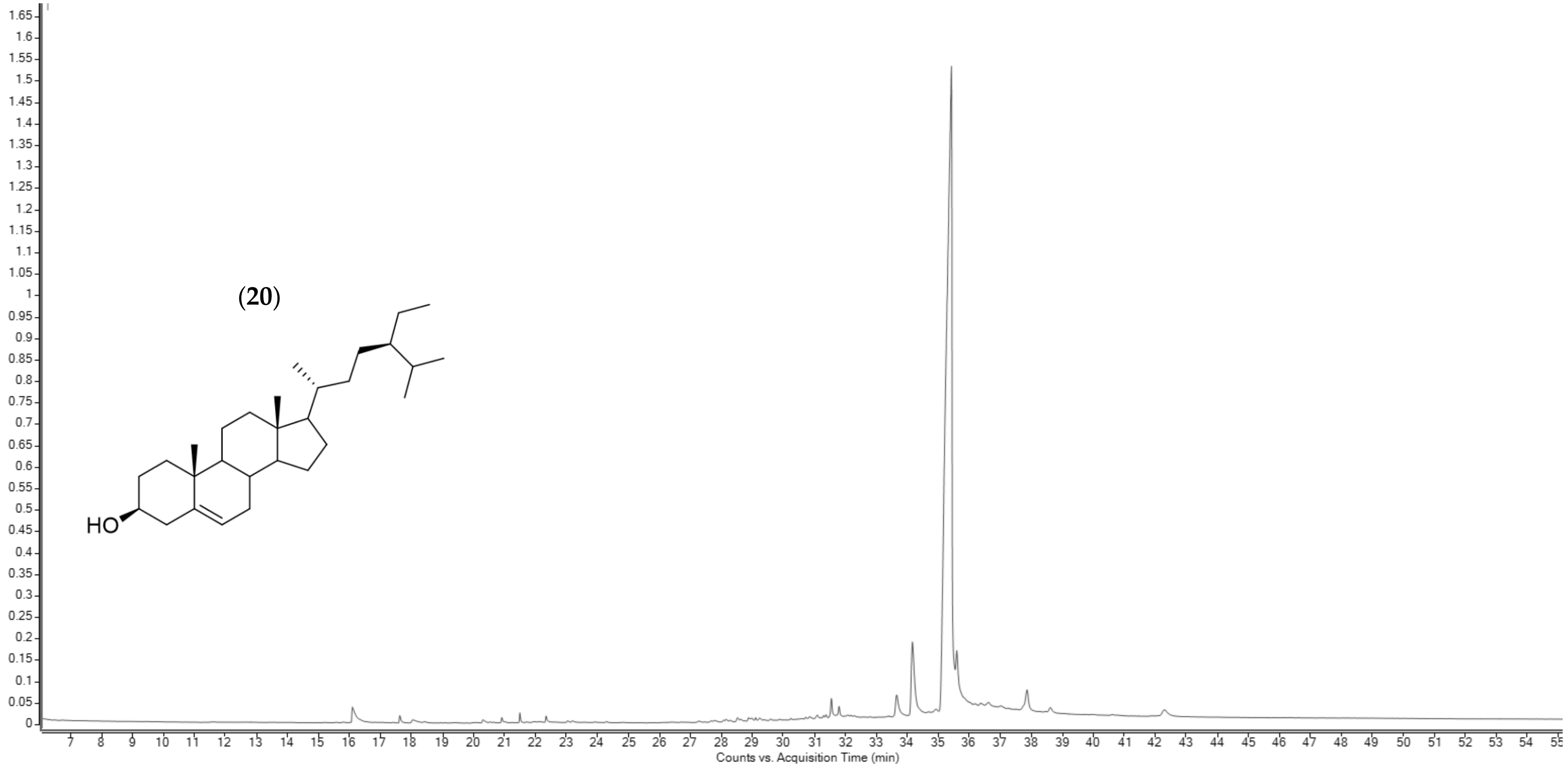

Figure S4. GC-MS Chromatogram of NPM 4 group corresponding to dehydrodiosgenin (**21**) RT=31.55 minutes and stigmasterol (**22**) RT=35.24 minutes.

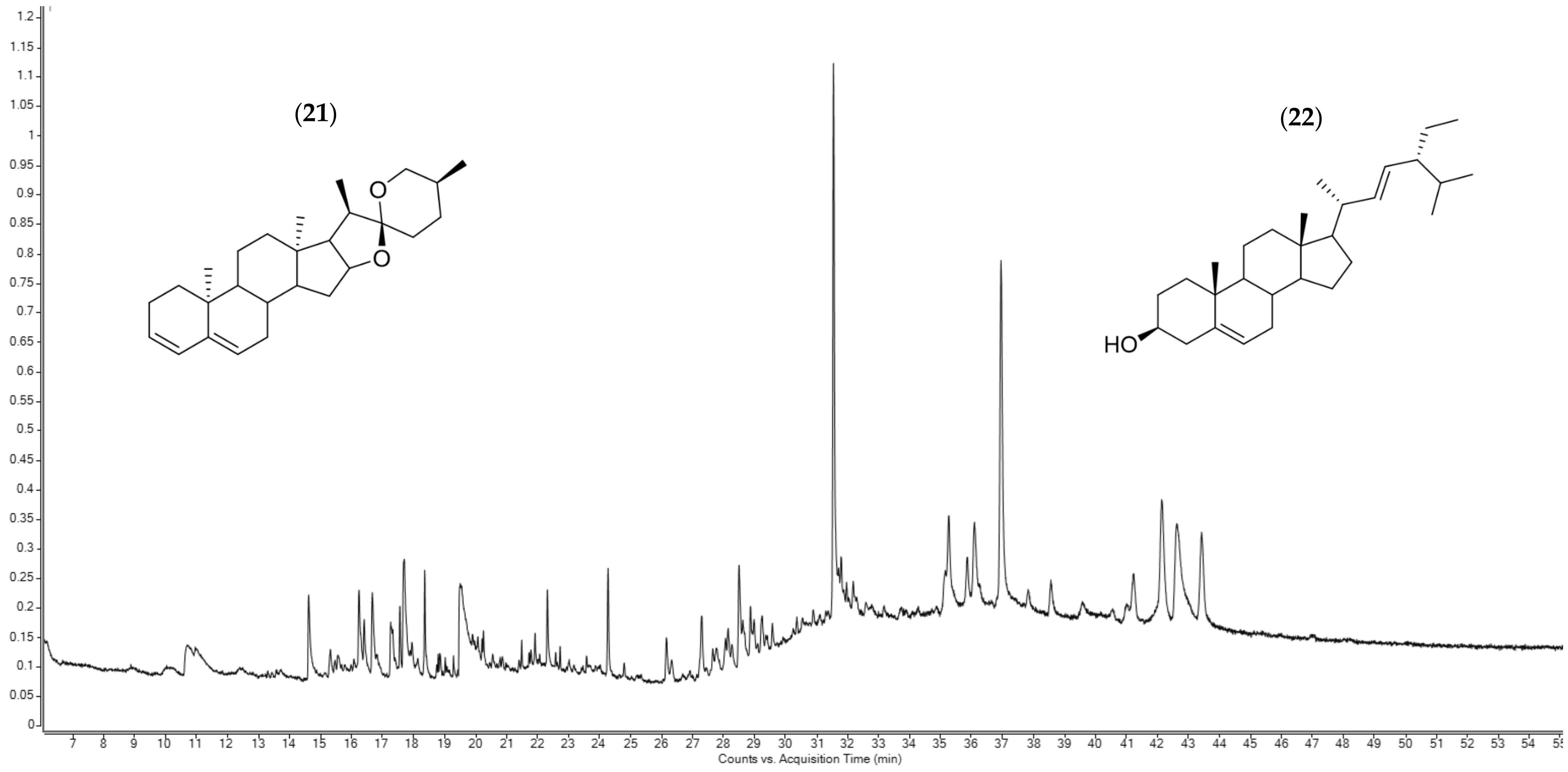

Figure S5. GC-MS Chromatogram of NPM 6 group corresponding to scopoletin (**23**) RT=19.26 minutes.

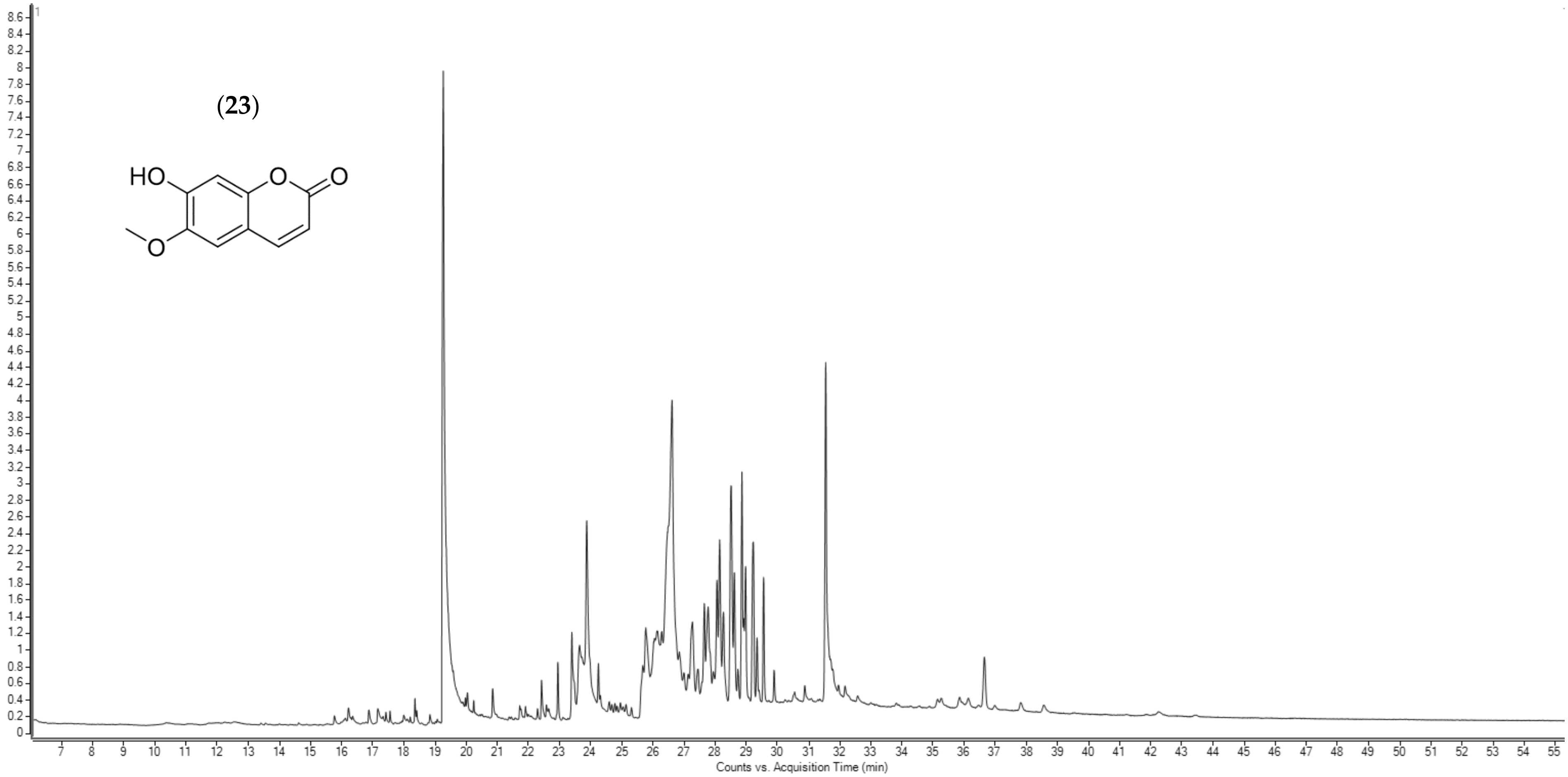

Figure 6S. HPLC chromatogram of the NPM-40-2 fraction containing the compound yatein (**24**) RT = 13.57 minutes. 250 nm.

(**24**)

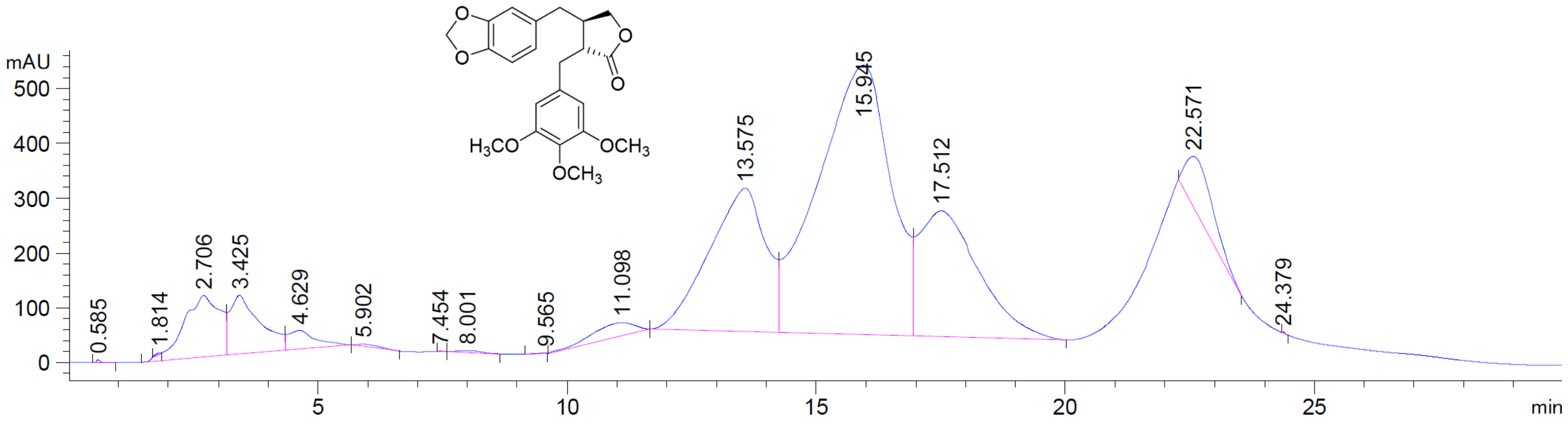

Figure S7. HPLC chromatogram of the NPM-40-3 and NPM-40-4 fractions containing the compounds 7′8-dehydropodophyllotoxin (**25**) RT= 11.76 minutes and acetyl podophyllotoxin (**26**) tR= 16.24 minutes, 250 nm.

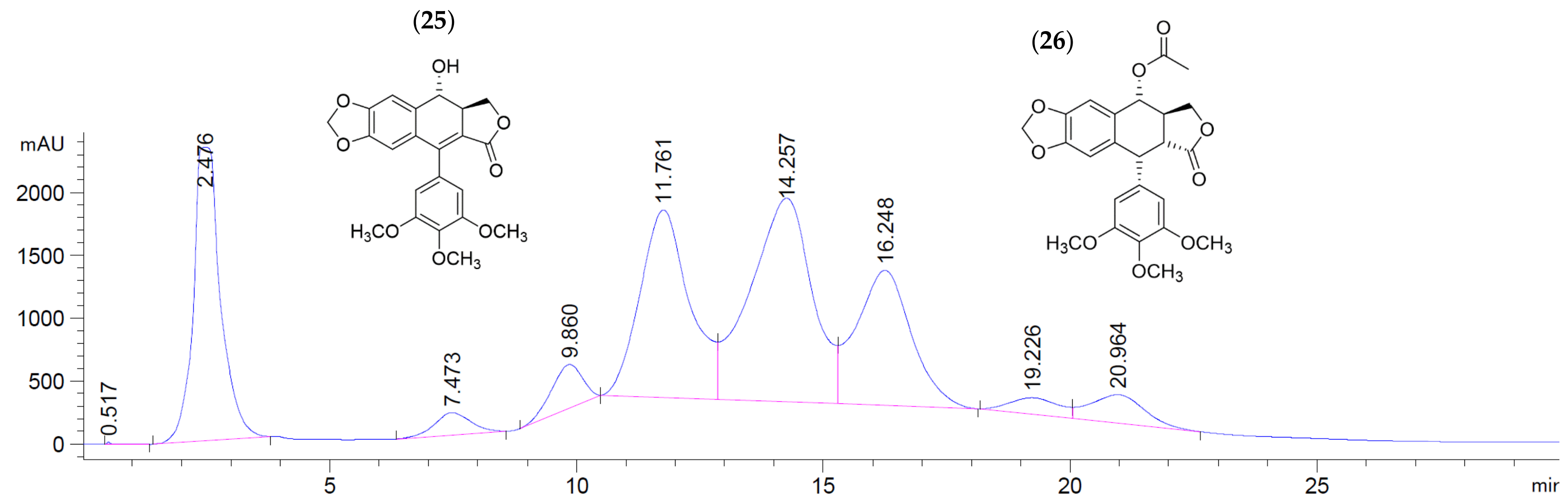

Supplement: Supplementary file 1 [file plants-13-01622-s001.zip › plants-2981348-supplementary.pdf]
